# Editorial

**Published:** 2011-08-16

**Authors:** Usha Mohan Das

**Affiliations:** Editor-in-Chief

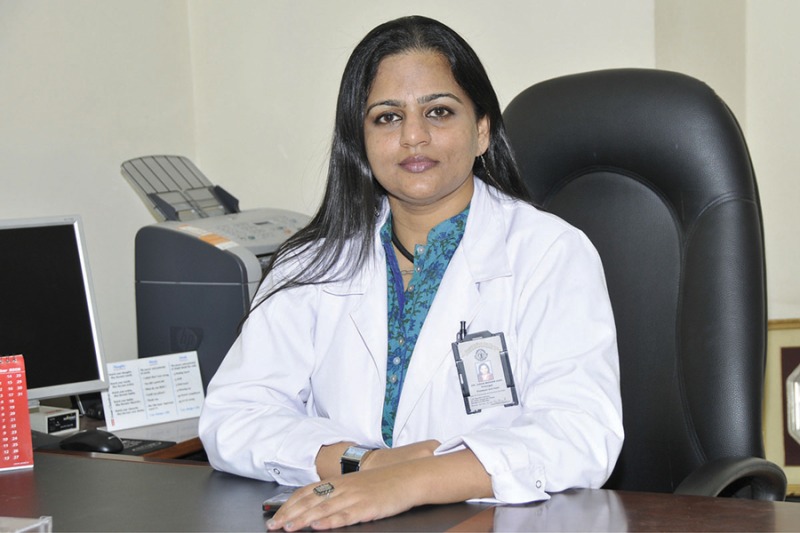



**Quantity *vs* Quality**

The editorial team and I are proud that IJCPD has been growing continuously. Indeed there is a tremendous scope for refinement and evolution.

Making publications mandatory for promotion and tenure of teachers in Dental Institutions in India surely sounds like agreat idea, but this rule has resulted in a desperate rat race for publishing that too in almost any journal or magazine one can lay hands on.

Ironically the dictum today is publish or perish!

It has become more of a number game!...An issue of quantity versus quality...An issue of time versus ethics!

One of the main problems facing publishing is a decline in ethics as a result of pressure to publish! The number of authors sometimes exceed the scientific content of the article itself. Everyone claims that they have made a substantial contribution to the study. Such trends may lead to student-faculty conflicts in future, besides a lot many other problems. Pressure to publish just sows seeds of fear of not being able to cope, as a result of which is born a phenomenon...Plagiarism.

The trend today is to learn quickly that finding and manipulating data on the Internet is a valuable skill! With the wealth of information available online, the production of original analysis and interpretation may seem like “busy work” compared to finding the best or most obscure sources. In fact the real skills students need to learn are interpretation and analysis ― how to process the information they find and not look fo easy searching options.

Let us not crack up under the burden of peer pressure or try complying with just the number of publications being demanded, instead, insist on quality and ethical practices. Authors, while submitting, should take all reasonable steps to ensure the quality of the material they publish, recognizing the different aims and standards of each journal.

I take this opportunity to dispel the myth that one would perish if they do not publish! The quality of a publication matters the most. It is a result of high intent, sincere effort, intelligent direction and skillful execution inspite of many alternatives.

**Usha Mohan Das**

Editor-in-Chief

e-mail: ushymohandas@gmail.com

